# Variants in the *BACH2* and *CLEC16A* gene might be associated with susceptibility to insulin‐triggered type 1 diabetes

**DOI:** 10.1111/jdi.13057

**Published:** 2019-05-14

**Authors:** Hiroshi Onuma, Ryoichi Kawamura, Yasuharu Tabara, Masakatsu Yamashita, Jun Ohashi, Eiji Kawasaki, Akihisa Imagawa, Yuya Yamada, Daisuke Chujo, Kenji Takahashi, Tadashi Suehiro, Yasunori Takata, Haruhiko Osawa, Hideichi Makino

**Affiliations:** ^1^ Department of Diabetes and Molecular Genetics Ehime University Graduate School of Medicine To‐on Ehime Japan; ^2^ Department of Diabetes, Endocrine and Metabolic Disease Tokyo Women's Medical University Yachiyo Medical Center Yachiyo Chiba Japan; ^3^ Center for Genomic Medicine Kyoto University Graduate School of Medicine Kyoto Japan; ^4^ Department of Immunology Ehime University Graduate School of Medicine To‐on Ehime Japan; ^5^ Department of Biological Sciences Graduate School of Science The University of Tokyo Tokyo Japan; ^6^ Department of Diabetes and Endocrinology Shin‐Koga Hospital Kurume Fukuoka Japan; ^7^ Department of Internal Medicine (I) Osaka Medical College Takatsuki Osaka Japan; ^8^ Department of Endocrinology and Metabolism Sumitomo Hospital Osaka Japan; ^9^ Department of Diabetes, Endocrinology and Metabolism National Center for Global Health and Medicine Tokyo Japan; ^10^ Department of Internal Medicine Diabetes Division Kurashiki Central Hospital Kurashiki Okayama Japan; ^11^ Department of Diabetes Kochi Takasu Hospital Kochi Kochi Japan; ^12^ Shiraishi Hospital Diabetes Center Imabari Ehime Japan

**Keywords:** *BACH2*, *CLEC16A*, Insulin‐triggered type 1 diabetes

## Abstract

**Aim/Introduction:**

Insulin administration was found to trigger type 1 diabetes in six Japanese type 2 diabetes patients with type 1 diabetes high‐risk human leukocyte antigen class II and the class I allele of the insulin gene variable number tandem repeat genotype. The objective of the present study was to assess the contribution of non‐human leukocyte antigen single‐nucleotide polymorphisms (SNPs) to the risk of developing insulin‐triggered type 1 diabetes.

**Materials and Methods:**

We genotyped 13 type 1 diabetes susceptible SNPs in six patients and compared them with those in Japanese controls (Hap Map3‐JPT). The SNPs that showed statistically significant results were further analyzed using non‐diabetic control participants and participants with type 2 diabetes at the Ehime University Hospital.

**Results:**

The risk allele frequency of *BACH2* rs3757247 in the six patients was significantly more frequent than that in 86 Japanese controls (*P *=* *0.038). No significant difference in the allele frequency was observed in the other SNPs. This result was confirmed by the findings that the risk allele frequency of *BACH2* in the six patients was significantly higher than that in the non‐diabetic control participants (*n* = 179) and type 2 diabetes with or without insulin treatment (*n* = 154 or *n* = 152; *P *=* *0.035, 0.034 or 0.037, respectively). Despite being statistically not significant, the six patients were all homozygous for the *CLEC16A* rs12708716 risk allele and five were homozygous for the *CLEC16A* rs2903692 risk allele.

**Conclusions:**

In addition to type 1 diabetes high‐risk human leukocyte antigen class II and the class I allele of the insulin gene variable number tandem repeat genotype, the possibility that the risk variants of *BACH2* and *CLEC16A* could contribute to the development of insulin‐triggered type 1 diabetes cannot be excluded.

## Introduction

Type 1 diabetes is an autoimmune disease characterized by an insulin deficiency resulting from the destruction of pancreatic β‐cells^1^. CD4^+^ and CD8^+^ T cells play an important role in the pathogenesis of the disease. Among the autoantigens, insulin plays the most important role in the type 1 diabetes process. In fact, insulin autoantibodies can be detected in children at an early age^2^, ^3^. Nakayama *et al*.^4^ and Kent *et al*.^5^ proposed that insulin itself is the primary autoantigen for autoimmune type 1 diabetes in mice and humans, suggesting that insulin is an essential autoantigen in type 1 diabetes in humans as well as mice. Based on these findings, an intervention trial using oral insulin for human type 1 diabetes was carried out. Recently, TrialNet subsequent to DPT‐1 failed to meet the primary end‐point of delaying or preventing diabetes onset^6^. Contrary to the intervention trial, it has been reported that the immunization of insulin can induce insulitis, a hallmark of autoimmune type 1 diabetes^7^, ^8^. In addition, we previously reported that insulin administration might trigger type 1 diabetes in Japanese type 2 diabetes patients with type 1 diabetes high‐risk human leukocyte antigen (HLA) class II (IDDM1) and the class I allele of the insulin gene variable number tandem repeat genotype (IDDM2)^9^, ^10^. The class I allele affects the expression of the insulin gene and the selection of insulin‐specific autoreactive T cells in the thymus^11^. We suspect that central tolerance might be less efficient due to IDDM1 and IDDM2, resulting in an increase in susceptibility to the T‐cell‐mediated immune response to insulin. In addition, it is also possible that peripheral tolerance might be involved in the pathogenesis of insulin‐triggered type 1 diabetes.

Environmental, as well as genetic, factors can contribute to the pathogenesis of type 1 diabetes. The incidence rates for type 1 diabetes varies among ethnic groups, and in contrast to Caucasians, Japanese individuals have one of the lowest levels of incidence, which might be due to the difference in IDDM1^12^, ^13^. In contrast, as has been reported previously^10^, IDDM2 is more frequent in the Japanese population^14^ than in Caucasians^15^. Genome‐wide association studies using large case–control cohorts of European ancestry revealed the existence of >60 susceptibility loci for type 1 diabetes^16^. However, their individual contribution to type 1 diabetes risk is low and their function constitutes an issue that remains to be solved. Interestingly, type 1 diabetes risk loci show concordant overlap with other seropositive autoimmune diseases, whereas discordant association is more common in seronegative autoimmune diseases^16^. Furthermore, >90% of disease‐associated single nucleotide polymorphisms (SNPs) map within the non‐coding regions of the genome, suggesting a regulatory role^16^. As most variants contribute to only modest effects to disease risk, several studies have evaluated a combination of variants. In The Environmental Determinants of Diabetes in the Young study, eight SNPs were reported to be significantly associated with the development of islet autoimmunity^17^. The type 1 diabetes TrialNet Study Group recently reported that five SNPs are associated with an increased risk of progression from islet autoantibody positivity to diabetes^18^.

However, it has been difficult to carry out genome‐wide association studies in Japanese individuals with type 1 diabetes because of its low incidence rate. Ikegami *et al*.^19^ assembled a multicenter study group in order to carry out large‐scale studies of Japanese individuals, and confirmed several type 1 diabetes susceptible loci that were also reported in Caucasians. Ayabe *et al*.^20^ genotyped 63 susceptibility variants in >400 Japanese childhood‐onset autoimmune type 1 diabetes patients, and found 10 risk alleles that were significantly more frequent in type 1 diabetes patients than in non‐diabetic individuals. In the present study, we analyzed 13 type 1 diabetes susceptible SNPs, and compared them with those in controls and patients with type 2 diabetes clarify the genetic background of insulin‐triggered type 1 diabetes patients.

## Methods

### Participants

Six patients with insulin‐triggered type 1 diabetes were the patients that were reported in our previous study^10^. Non‐diabetic control participants and participants with type 2 diabetes were recruited from the Ehime University Hospital in Japan. Diabetes mellitus was diagnosed according to the 1998 American Diabetes Association criteria. Non‐diabetic control participants were selected based on the absence of a personal and familial history of diabetes in their first‐degree relatives, as well as either normal glucose tolerance based on a 75‐g oral glucose tolerance test or glycated hemoglobin levels <5.6% with fasting plasma glucose levels <110 mg/dL. All patients and non‐diabetic participants were informed of the purpose of the study, and written consent was obtained from each participant. The study was approved by the ethics committee of the Ehime University Hospital Graduate School of Medicine, and was carried out in accordance with the Declaration of Helsinki.

### Genotyping

Genomic DNA was extracted from peripheral blood. A total of 13 type 1 diabetes susceptible SNPs were selected from the genes, which are thought to be immune‐ or pancreatic islet‐related^21^. The *PTPN22* rs2488457, *IL2RA* rs706778, *IL2RA* rs3118470, *ERBB3* rs2292239, *PTPN2* rs1893217, *CLEC16A* rs12708716, *CLEC16A* rs2903692, *CTLA4* rs3087243, *IFIH1* rs1990760, *CD226* rs763361, *UBASH3A* rs9976767, *BACH2* rs3757247 and *BACH2* rs11755527 were analyzed by a TaqMan probe assay (Applied Biosystems Co., Ltd., Foster City, CA, USA) using commercially available primers and probes purchased from the Assay‐on‐Demand system. An ABI PRISM 7900HT sequence detector (Applied Biosystems) was used to measure the fluorescence levels of the polymerase chain reaction products. The HLA class I and II genotypes were determined as reported previously^9^.

### Statistical analysis

The differences in risk allele frequency of type 1 diabetes susceptibility genes between Japanese controls and the six patients with insulin‐triggered type 1 diabetes was determined by Fisher's exact probability test using HapMap3‐JPT (http://grch37.ensembl.org/Homo_sapiens/Info/Index) data as control Japanese with the js‐STAR version 8.0.1j (Nappa, Tokyo, Japan). The differences in risk allele frequency between non‐diabetic control participants or participants with type 2 diabetes at the Ehime University hospital and the six patients (insulin‐triggered type 1 diabetes) were determined by Fisher's exact probability test.

## Results

### T allele of *BACH2* rs3757247 in the six insulin‐triggered type 1 diabetes was more frequent than that in control Japanese participants (HapMap3‐JPT)

In a previous study, we reported on an analysis of the characteristics of the six patients with insulin‐triggered type 1 diabetes 10. The six patients were composed of four men and two women. The mean age at onset of type 1 diabetes was 59.5 ± 12.8 years. The mean duration of type 2 diabetes until the development of type 1 diabetes was 16.8 ± 11.8 years. The mean body mass index was 24.6 ± 5.0 kg/m^2^. Insulin treatment was initiated because of deteriorated glycemic control (glycated hemoglobin 9–11%) in all patients. The mean duration of insulin administration until the onset of type 1 diabetes was 7.7 ± 6.1 months. We first genotyped 13 previously reported SNPs associated with type 1 diabetes described in the Methods section in the six patients with insulin‐triggered type 1 diabetes and compared them with those in 86 control Japanese participants using HapMap3‐JPT data (http://grch37.ensembl.org/Homo_sapiens/Info/Index). The results for the genotyping of each SNP in the six patients with insulin‐triggered type 1 diabetes are shown in Table 1. The risk allele (T‐allele) frequency of *BACH2* rs3757247 in the six patients with insulin‐triggered type 1 diabetes was 0.75, and was significantly more frequent than that in 86 control Japanese participants (0.43; *P *=* *0.0382). Aside from *BACH2* rs3757247, no significant difference in the allele frequency was observed in the other SNPs.

**Table 1:** Genotype of 13 single‐nucleotide polymorphisms of the type 1 diabetes susceptibility gene in the six patients with insulin‐triggered type 1 diabetes

Nearby gene	SNP	Risk allele	Non‐risk allele	Risk allele frequency (Hap Map3‐JPT)	Case 1	Case 2	Case 3	Case 4	Case 5	Case 6	*P*
*PTPN22*	rs2488457	C	G	0.57	G/G	G/G	G/G	G/C	C/C	G/C	0.563
*IL2RA*	rs706778	T	C	0.48	T/T	T/C	T/C	T/C	C/C	T/C	1
*IL2RA*	rs3118470	C	T	0.44	C/C	T/C	T/C	T/C	T/T	T/C	0.769
*ERBB3*	rs2292239	A	C	0.24	C/C	A/C	A/A	C/C	C/C	C/C	1
*PTPN2*	rs1893217	G	A	0.1	A/A	A/A	A/A	A/A	G/A	A/A	1
*CLEC16A*	rs12708716	A	G	0.83	A/A	A/A	A/A	A/A	A/A	A/A	0.220
*CLEC16A*	rs2903692	G	A	0.83	G/G	G/G	G/A	G/G	G/G	G/G	0.694
*CTLA4*	rs3087243	G	A	0.73	G/A	G/A	G/A	G/G	G/A	G/G	0.740
*IFIH1*	rs1990760	T	C	0.25	C/C	T/C	C/C	C/C	T/C	T/C	1
*CD226*	rs763361	T	C	0.47	T/C	T/C	C/C	C/C	C/C	T/T	0.551
*UBASH3A*	rs9976767	G	A	0.38	G/A	A/A	A/A	G/A	A/A	A/A	0.215
*BACH2*	rs3757247	T	C	0.43	T/T	T/C	T/T	T/C	T/C	T/T	0.038
*BACH2*	rs11755527	G	C	0.378	G/G	G/C	G/G	G/C	G/C	G/C	0.066

*P* refers to the difference of risk allele frequency between control Japanese participants (*n* = 172) and the six patients (insulin‐triggered type 1 diabetes) were determined using HapMap3‐JPT data (*n* = 86) by Fisher's exact probability test (two‐sided test). SNP, single‐nucleotide polymorphism.

### T allele of *BACH2* rs3757247 in insulin‐triggered type 1 diabetes was more frequent than that in non‐diabetic control participants and participants with type 2 diabetes

We further genotyped the *BACH2* rs3757247, the risk allele frequency of which is significantly different in risk allele frequency between the six patients with insulin‐triggered type 1 diabetes and 86 control Japanese participants using HapMap3‐JPT data in non‐diabetic control participants (*n* = 179), type 2 diabetes patients with insulin treatment (*n* = 154), and type 2 diabetes patients without insulin treatment (*n* = 152) at the Ehime University Hospital (Table 2). The frequency of the T allele in non‐diabetic control participants, type 2 diabetes patients with insulin treatment and type 2 diabetes patients without insulin treatment was found to be 0.42, 0.42 and 0.43, respectively. The T allele frequency of *BACH2* in the six patients with insulin‐triggered type 1 diabetes (0.75) was found to be significantly higher than that in non‐diabetic control participants and type 2 diabetes patients with and without insulin treatment (*P *=* *0.035, 0.034 and 0.037, respectively). As for the genotype frequency, there was a trend in the same direction, but did not reach statistical significance.

**Table 2:** Genotypes and allele frequency of *BACH2* rs3757247 in the six patients with insulin‐triggered type 1 diabetes compared with non‐diabetic controls and type 2 diabetes patients with or without insulin therapy

BACH2 rs3757247	*n*	Genotype			*P*	Allele		*P*
		T/T	C/T	C/C		T	C	
Insulin‐triggered T1D	6	3	3	0	*	9 (75%)	3 (25%)	*
Non‐diabetic control	179	33	85	61	0.0665	151 (42.2%)	207 (57.8%)	0.0352
T2D with insulin therapy	154	27	74	53	0.063	128 (41.5%)	180 (58.5%)	0.0338
T2D without insulin therapy	152	31	68	53	0.0766	130 (42.7%)	174 (57.3%)	0.0367

*P*: the difference of genotype and risk allele frequency between the six patients (insulin‐triggered type 1 diabetes) and non‐diabetic control participants and patients with type 2 diabetes were determined by Fisher's exact probability test (two‐sided test). *, reference; T1D, type 1 diabetes; T2D, type 2 diabetes.

### Total risk allele count in each SNP in the six patients with insulin‐triggered type 1 diabetes

**Figure 1:**
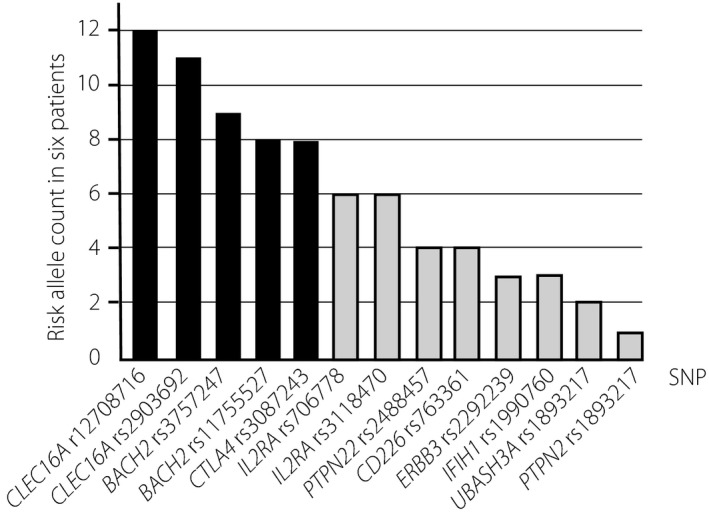
Total risk allele count in each single‐nucleotide polymorphism (SNP) of the six patients. The number of total risk alleles in each type 1 diabetes susceptible SNP of the six patients were counted. The black bars show the SNP having one or two risk alleles in each patient with insulin‐triggered type 1 diabetes.

The total risk allele count in each SNP for the six patients with insulin‐triggered type 1 diabetes is shown in Figure 1. The black bar indicates the risk variant having one or two risk alleles in each patient with insulin‐triggered type 1 diabetes. The six patients with insulin‐triggered type 1 diabetes were all homozygous for the risk allele of *CLEC16A* rs12708716, and five patients were homozygous for the risk allele of *CLEC16A* rs2903692. The risk allele count for *BACH2* rs3757247 was nine, and that of *BACH2* rs11755527and *CTLA4* rs3087243 were both eight, respectively.

### HLA class II and class I genotypes in the six patients with insulin‐triggered type 1 diabetes

As reported previously^10^, all of the patients had type 1 diabetes susceptible HLA class II haplotypes, as reported for Japanese, such as DRB1*04:05‐DQB1*04:01 and/or DRB1*09:01‐DQB1*03:03 (Table 3). Three patients had type 1 diabetes susceptible DPB1*02:01 allele, as reported in Japanese patients with type 1 diabetes^12^, ^22^. Regarding the HLA class I alleles, five patients (patients 1, 3, 4, 5 and 6) had type 1 diabetes susceptible alleles – A*24:02, B*40:06 or B*54:01 and Cw*01:02 or Cw*08:01 – as has been previously reported in Japanese patients with type 1 diabetes 12, 22. Patient 2 had none of these type 1 diabetes susceptible alleles, but his clinical characteristics were similar to those of the other five patients.

**Table 3:** Human leukocyte antigen‐class II and I genotypes in the six patients with insulin‐triggered type 1 diabetes

HLA	Patient 1	Patient 2	Patient 3	Patient 4	Patient 5	Patient 6
DRB1	*04:05/*09:05	*08:03/*09:01	*04:05/*04:05	*01:01/*09:01	*04:05/*14:07	*09:01/*09:01
DQB1	*03:03/*04:01	*03:01/*03:03	*04:01/*04:01	*03:03/*05:01	*04:01/*05:02	*03:03/*03:03
DPB1	*05:01/*05:01	*05:01/*14:01	*02:01/*09:01	*02:01/*02:01	*05:01/*14:01	*02:01/*05:01
A	*02:01/*02:01	*02:01/*02:01	*11:01/*24:02	*24:02/*26:02	*24:02/*31:01	*24:02/*26:02
B	*40:01/*54:01	*38:02/*40:01	*15:01/*54:01	*40:06/*52:01	*48:01/*54:01	*40:06/*51:01
C	Cw*01:02/*03:04	Cw*03:04/*07:02	Cw*01:02/*04:01	Cw*08:01/*12:02	Cw*01:02/*08:03	Cw*08:01/*14:02

HLA, human leukocyte antigen.

## Discussion

The findings reported herein show that *BACH2* rs3757247 was significantly more frequent in the six patients with insulin‐triggered type 1 diabetes than in non‐diabetic control participants and type 2 diabetes patients with or without insulin treatment. *BACH2* is a transcription factor associated with a super‐enhancer, and the gene is located on human chromosome 6q15^23^. The SNPs, rs3757247^24^ and rs11755527^25^, which are in tight linkage disequilibrium (LD), have been reported to be associated with type 1 diabetes in Caucasian individuals. Ayabe *et al*.^20^ confirmed the association of rs3757247 in Japanese individuals with childhood‐onset type 1 diabetes. Several SNPs in LD in the *BACH2* locus are associated with a variety of autoimmune diseases^23^, suggesting that its function might be related to fundamental immune regulation.

In fact, using *Bach2* knockout mice, Roychoudhuri *et al*.^26^ showed a fatal inflammation, especially in the lung and colon, with anti‐nuclear autoantibodies when the mice were several months old. They suggested that *Bach2* is a key regulator of the generation of regulatory T cells (Tregs) and effector T‐cell differentiation, processes that prevent the development of inflammatory diseases by controlling the balance between tolerance and immunity. Afzali *et al*.^27^ reported on a heterozygous point mutation in the *BACH2* locus in the two families who showed autoimmune gastrointestinal disease, recurrent respiratory tract infections and an immunoglobulin deficiency. They also proposed that *BACH2* is a key regulator of the human adaptive immune system, which is critical to the maintenance of Tregs formation and B‐cell maturation. Furthermore, they also proposed that in super‐enhancer‐associated genes, such as the *BACH2* gene, small changes in expression level might lead to amplified changes in their associated network, resulting in significant pathological changes.

**Figure 2:**
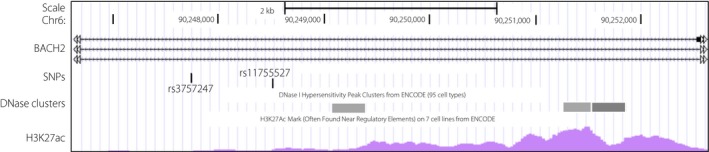
Both single‐nucleotide polymorphisms (SNPs), rs3757247 and rs11755527, might be located in the enhancer region of the BACH2 gene (UCSC Genome Browser on Human Assembly: GRCh38/hg38). DNase clusters: DNase I Hypersensitivity Clusters from ENCODE (95 cell types) are shown as the grey boxes. H3K27ac: H3K27AC MARK (Often Found Near Regulatory Elements) on T‐cell lines from ENCODE are shown as the pink waveform.

In a previous study, we observed typical insulitis in the islets of one patient, and insulin‐peptides‐reactive type 1 T helper cells, as well as an insulin allergy or a high titer of insulin antibody in several other patients^9^, ^10^. Although rs3757247 is an intronic variant that is located in the intron 2 of the *BACH2* locus, it is possible that slight changes in the expression level might lead to significant alterations in Tregs function, resulting in a T‐cell‐mediated immune response to insulin. Indeed, the histone H3K27 hyperacetylation region and DNase I hypersensitive sites have been reported in the proximity region of the rs3757247 and rs11755527 (UCSC Genome Browser on Human Assembly: GRCh38/hg38; Figure 2). It is well established that the active enhancers can be identified by enrichment of both H3K27 and hypersensitive sites for DNase I^28^, ^29^. Both SNPs might be located in the enhancer region of the BACH2 gene and could, therefore, have an influence on *BACH2* transcription in Tregs.

As shown in Figure 1, all six of the patients were homozygous for the risk allele of *CLEC16A* rs12708716, and five patients were homozygous for the risk allele of *CLEC16A* rs2903692, suggesting a causal relationship with the present patients, despite the fact that the data were not statistically significant. *CLEC16A* is an atypical C‐type lectin, and the gene is located on human chromosome 16p13^30^. SNPs, rs2903692 (intron 22) and rs12708716 (intron 19), which are in tight LD have been reported to be associated with Caucasian subjects with type 1 diabetes 31, 32, 33. Awata *et al*.^34^ confirmed the association of rs2903692 in Japanese subjects with type 1 diabetes. Several SNPs in LD in the *CLEC16A* locus (formerly referred to as *KIAA0350*) are associated with other autoimmune diseases^35^. Therefore, its function might be related to fundamental immune regulation, as has been suggested for the *BACH2* gene.

Schuster *et al*.^35^ reported that *Clec16A* knockdown mice showed a diminished diabetogenicity of non‐obese diabetic T cells, which might be due to an alteration in the thymic epithelial cell (TEC) stimulation of thymocytes owing to impaired autophagy. As the complete absence of *Atg5*, which is an essential component of autophagosome formation, was found to be lethal in neonate mice, Nedjic *et al*.^36^ transplanted embryonic *Atg5*‐deficient thymi under the capsule of a normal adult recipient. They found that the selection of certain major histocompatibility complex‐restricted T‐cell specificities and severe inflammation in the colon and liver had been altered, and concluded that autophagy in TEC contributes to T‐cell selection and is essential for the generation of a self‐tolerant T‐cell repertoire. Although *CLEC16A* in TEC might not have as strong an effect on its autophagy as that of *ATG5*, it is possible that the intronic variants of *CLEC16A* might cause slight changes in the autophagy of TEC, resulting in a less efficient deletion of insulin‐specific autoreactive T cells.

Until now, insulin‐triggered type 1 diabetes has been reported only in Japanese individuals, but not from other countries. In a previous study, we reported a significantly higher odds ratio of IDDM2 in Japanese patients with type 1 diabetes than in Caucasian patients with type 1 diabetes 10. Ayabe *et al*.^20^ recently reported a high odds ratio for IDDM2 using SNP rs689 in Japanese patients with type 1 diabetes, which prompted us to again compare the odds ratio for the rs689 genotype between Japanese and Caucasian patients with type 1 diabetes^37^, and the results are shown in Table 4. We confirmed a significantly higher odds ratio of the IDDM2 genotype in Japanese patients with type 1 diabetes compared with those of Caucasian patients with type 1 diabetes (*P* = 0.042), indicating that Japanese individuals might be more sensitive to insulin autoimmunity than Caucasians. In fact, insulin‐triggered type 1 diabetes, as well as insulin autoimmune syndrome, has been reported exclusively in Japanese individuals^10^.

**Table 4:** Odds ratios for the association between *INS* rs689 and type 1 diabetes in Japanese and Caucasian individuals

	Genotype	Type 1 diabetes	Non‐diabetic control	OR (95% CI)
Japanese (Ayabe *et al*.^20^)		(*n* = 852)	(*n* = 910)	
	I/I	847(99.4%)	881 (96.8%)	5.58 (2.15–14.47)
	I/III or III/III	5 (0.6%)	29 (3.2%)	
Caucasian (Bjørnvold *et al*.^37^)		(*n* = 1,251)	(*n* = 1,413)	
	I/I	903 (72.2)	779 (55.1%)	2.11(1.80–2.48)
	I/III or III/III	348 (27.8%)	634 (44.9%)	

In Ayabe's study, the T allele in *INS*689 was reported as a risk allele (I), which corresponded to major alleles in Japanese individuals. CI, confidence interval; OR, odds ratio.

In conclusion, the risk variants of *CLEC16A*, as well as IDDM1 and IDDM2, could result in a less efficient deletion of insulin‐specific autoreactive T cells in the thymus. As the variant of *BACH2* could have a slightly altered Tregs function, it could not make an adjustment for the derangement of insulin‐specific autoreactive T cells in the periphery, resulting in the development of insulin‐triggered type 1 diabetes. However, as direct evidence for the function of these variants was lacking in the present study, further work will be required to clarify the pathological mechanism responsible for the present patients. Furthermore, as the present study only included a very small sample size, it will be necessary to confirm the findings in follow‐up studies with additional insulin‐triggered type 1 diabetes patients.

## Disclosure

The authors declare no conflict of interest.
